# Entity recognition in the biomedical domain using a hybrid approach

**DOI:** 10.1186/s13326-017-0157-6

**Published:** 2017-11-09

**Authors:** Marco Basaldella, Lenz Furrer, Carlo Tasso, Fabio Rinaldi

**Affiliations:** 1 Università degli Studi di Udine, Via delle Scienze 208, Udine, 33100 Italy; 2 University of Zurich, Institute of Computational Linguistics and Swiss Institute of Bioinformatics, Andreasstrasse 15, Zürich, CH-8050 Switzerland

**Keywords:** Named entity recognition, Text mining, Machine learning, Natural language processing

## Abstract

**Background:**

This article describes a high-recall, high-precision approach for the extraction of biomedical entities from scientific articles.

**Method:**

The approach uses a two-stage pipeline, combining a dictionary-based entity recognizer with a machine-learning classifier. First, the OGER entity recognizer, which has a bias towards high recall, annotates the terms that appear in selected domain ontologies. Subsequently, the Distiller framework uses this information as a feature for a machine learning algorithm to select the relevant entities only. For this step, we compare two different supervised machine-learning algorithms: Conditional Random Fields and Neural Networks.

**Results:**

In an in-domain evaluation using the CRAFT corpus, we test the performance of the combined systems when recognizing chemicals, cell types, cellular components, biological processes, molecular functions, organisms, proteins, and biological sequences. Our best system combines dictionary-based candidate generation with Neural-Network-based filtering. It achieves an overall precision of 86% at a recall of 60% on the named entity recognition task, and a precision of 51% at a recall of 49% on the concept recognition task.

**Conclusion:**

These results are to our knowledge the best reported so far in this particular task.

## Background

The scientific community in the biomedical domain is a vibrant community, producing a large amount of scientific findings in the form of data, publications, reports, and so on, each year, making it difficult for scholars to find the right information in this large sea of knowledge.

To tackle this problem, researchers have developed different text mining techniques with the goal of detecting the relevant information for the intended purpose. This paper’s focus is the technique called Named Entity Recognition (herein NER), which solves the problem of detecting terms belonging to a limited set of predefined entity types.

NER can be performed on both “generic” documents, to recognize concepts like *person*, *date* or *location*, or on technical documents, to recognize concepts like *cells*, *diseases* or *proteins*. NER can be used by itself, with the goal of recognizing the mere *presence* of a term in a certain portion of the text, or as a preliminary stage for Concept Recognition (CR), also known as Entity Linking or Normalization, where the term is not only recognized but also linked to a terminological resource, such as an ontology, through the use of a unique identifier [1].

NER can be solved using several techniques:

- Using manual, hand-written rules. A group of experts develops these rules using domain knowledge. The rules typically rely on orthographic patterns, such as particular use of capitalization or punctuation. Even though rule-based systems can perform well if sufficient expert time is available for creating the rules, their maintenance requires repeated manual efforts, since the rules need to be revised or even entirely replaced whenever the system is adapted to new data (different entity types, another text collection). In the biomedical domain, plain rule-based approaches like [2] have become rare; however, they continue to be used in combination with other techniques, such as dictionary look-up [3, 4].
- Using dictionaries to recognize known entities. An automatic process looks for all the possible entities (possibly, all the words in a document) in one or more dictionaries (or ontologies, or databases, or gazetteers) of known entities. This method has the obvious drawback that new entities cannot be recognized, because they are not yet present in the dictionary. Pafilis et al. [5] and [6] use a dictionary-based approach for NER of species and chemicals, respectively.
- Using machine learning techniques. A machine learning method, like Support Vector Machines or Conditional Random Fields (herein CRF), can be trained to recognize entities in a fragment of text using a large set of pre-annotated documents as examples. If trained properly, a machine learning model can potentially recognize entities that are not yet inserted in dictionaries or ontologies. The drawback of this approach is the fact that training material is not always available for a certain domain, or if present, it may be unsatisfactory in terms of quality or size. Examples for a CRF-based approach are presented in [7, 8].
- Using a hybrid approach. Two or more of the previously mentioned approaches are used together to combine their strengths and, hopefully, overcome their weaknesses. For example, [9] and [10] successfully use a hybrid dictionary-machine learning approach.

This paper presents an extension of the hybrid solution introduced in [11]. In that paper, we present a hybrid dictionary-machine learning approach, where the dictionary stage, performed by OntoGene’s Entity Recognizer (OGER) [12, 13], generates a high recall, low precision set of all the possible entities that can be found in a document and then the machine learning stage, performed by Distiller [14], filters these entities trying to select only the relevant ones.

The aim of this work is to improve the system presented in [11] by exploring new techniques both for the dictionary and the machine-learning stage, in particular by replacing the original machine learning approach with one based on CRFs. We present these techniques, analyzing the new methods we introduced, and we evaluate them on the CRAFT corpus [15], a set of documents from the biomedical domain where the relevant concepts have been linked to several ontologies. Then, we compare the results obtained with the ones found in the literature, exploring the potential of using the system as a concept recognition pipeline.

## Methods

### CRAFT corpus

The Colorado Richly Annotated Full Text (CRAFT) corpus is a set of articles from the PubMed Central Open Access Subset [16], a part of the PubMed Central archive licensed under Creative Commons licenses, annotated with concepts pointing to several ontologies.

The corpus is composed of 67 annotated articles available in the public domain, plus 30 articles that have been annotated but are reserved for future competitions and have to date not been released.

The ontologies used in the corpus are:

- **ChEBI**: Chemical entities of Biological Interest [17], containing **chemical** names
- **CL**: Cell Ontology [18], containing **cell type** names
- **Entrez Gene** [19], containing **gene** names
- **GO**: Gene Ontology [20]. CRAFT provides two sub-ontologies, one for physical entities (**cellular components**, CC) and one for non-physical entities (**biological processes** and **molecular functions**, BPMF).
- **NCBI Taxonomy**: the US National Center for Biotechnology Information Taxonomy [21], containing names of **species** and other taxonomic ranks
- **PR**: Protein Ontology [22], containing **protein** names
- **SO**: Sequence Ontology [23], containing names of **biological sequence** features and attributes

In total, the available articles are annotated with over 100,000 concepts. Moreover, each of the 67 articles contains linguistic information, such as tokenized sentences, part-of-speech information, and dependency parse trees.

For our experiments, we used all terminology resources except for NCBI Entrez Gene. We decided to omit Entrez Gene from the evaluation against CRAFT for a number of reasons. For one, the distribution of the CRAFT corpus does not include a reference version (unlike all other terminologies); this means that we would have to use an up-to-date version of Entrez Gene, which potentially differs significantly from the version used in the annotation process. Secondly, Entrez Gene contains a large number of terms that overlap with frequent words of the general vocabulary (such as “was”, “and”, “this”), taking care of which requires considerable additional effort, such as manually creating blacklists. Furthermore, omitting Entrez Gene has been suggested earlier by other scholars (e.g. ([24], p. 8)).

We associated each (sub-)ontology with a single entity type. For NCBI Taxonomy, we regarded species and higher taxonomic ranks (genus, order, phylum etc.) from both cellular organisms and viruses to a common entity type “organism”. For the Gene Ontology, we followed CRAFT’s division into physical and non-physical entities, i.e. we distinguished “cellular components” from “biological processes/molecular functions”.

### OGER

The OntoGene group has developed an approach for biomedical entity recognition based on dictionary lookup and flexible matching. Their approach has been used in several competitive evaluations of biomedical text mining technologies, often obtaining top-ranked results [25–28]. Recently, the core parts of the pipeline have been implemented in a more efficient framework using Python [29] and are now developed under the name OGER (OntoGene’s Entity Recognizer). These improvements showed to be effective in the BioCreative V.5 shared task [30]: in the *technical interoperability and performance of annotation servers* (TIPS) task, our system achieved best results in four out of six evaluation metrics [31]. In the TIPS task, participants were asked to provide an on-line service for on-the-fly annotation of biomedical entities in given documents. The task’s goal was to investigate the feasibility of installing an inter-institutional annotation cluster (controlled by a *biomedical annotation metaserver*), therefore the evaluation was based on processing time and availability of the participating systems [32]. OGER achieved single first place in the speed measures (average response time, mean time per document volume) and shared first place in the stability measures (mean time between failures, mean time to repair).

OGER offers a flexible interface for performing dictionary-based NER. It accepts a range of input formats, e.g. PubMed Central full-text XML, gzip-compressed chunks of Medline abstracts as made available for download by PubMed, BioC XML [33], or simply plain text. It provides the annotated terms along with the corresponding identifiers either in a simple tab-separated text file, in brat’s standoff format [34], in BioC XML, or in a number of other, less common formats. It allows for easily plugging in additional components, such as alternative NLP preprocessing methods or postfiltering routines. We run an instance of OGER as a permanent web service which is accessible through an API and a web user interface [35].

For term matching, we used the terminology resources included in the CRAFT corpus. We extracted the relevant information from the various sources and converted it into a unified, non-hierarchical format, in order for it to be accepted by the annotation pipeline. For the format conversion, we used the back-end software of the Bio Term Hub [36], which is a meta-resource for biomedical terminological resources. Through a web interface [37], any user can obtain a customized dictionary, which is compiled on the fly from a number of curated, openly available terminology databases.

By concatenating the selected seven terminologies, we obtained a dictionary with 1.26 million terms pointing to 864,000 concept identifiers. Based on preliminary tests, we removed all entries with terms shorter than three characters or terms consisting of digits only; this reduced the number of entries by less than 0.1%. In OGER, the entries of the term dictionary were then preprocessed in the same way as the documents with respect to tokenization, stemming, and case sensitivity, as described below. Finally, the input documents were compared to the dictionary with an exact-match strategy.

OGER was configured to have a moderate bias towards recall, at the cost of precision. We chose this strategy, tailored to a greater number of false positives (i.e. lower precision) but less false negatives (i.e. greater recall), because in the overall architecture OGER’s output is filtered or used as a feature among many in the subsequent step, so we let the subsequent ML step decide which of the annotations produced by the dictionary step are actually useful.

After sentence splitting, the input documents were tokenized with a simple method based on character class: any contiguous sequence of either alphabetical or numerical characters was considered a token, whereas any other characters (punctuation and whitespace) were considered token boundaries and were ignored during the dictionary look-up. This lossy tokenization already has a normalizing effect, in that it collapses spelling variants which arise from inconsistent use of punctuation symbols. For example, the variants “SRC 1”, “SRC-1”, and “SRC1” were all conflated to the two-token sequence “SRC”, “1”. In a small evaluation on the training set, we verified that this results in a moderate improvement of overall recall (+ 2.7 percentage points) and worked particularly well for sequences (+ 3.5) and proteins (+ 8.4), while the effect on precision was negative, but smaller (− 2.1). A similar approach is described in [38], where the authors refer to it as “regularization”. All tokens were then converted to lowercase, except for acronyms that collide with a word from general language (e.g. “WAS”). We enforced a case-sensitive match in these cases by using a list of the most frequent English words. As a further normalization step, Greek letters were expanded to their letter name in Latin spelling, e.g. “ *α*” → “alpha”. Since both spellings are common in the biomedical literature, converting all occurrences to a canonical form allowed us to increase the number of matches. Finally, we applied stemming to all tokens except for the acronyms, using NLTK’s [39] implementation of the Lancaster stemmer [40]. We favored this algorithm over the more widely used Porter stemmer because of its greater strength, i.e. its higher amount of conflations produced, which increases the overlap between the dictionary and the documents, in line with our aim for higher recall.

As a tweaking step, we fine-tuned the above default configuration for the individual entity types. Based on their respective coverage in the training set, we adjusted the parameters as follows:

- **proteins** no stemming
- **sequences** less strict acronym filter (more cases of case-insensitive matching)
- **cells** always case-insensitive matching (even for acronyms)
- **cellular components** always case-insensitive matching

### Distiller

The Distiller framework [41] is an open source project which aims to build a flexible, extensible system for a variety of natural language processing tasks [14].

The main focus of the Distiller framework is the task of Automatic Keyphrase Extraction (herein AKE), which is the process of extracting *relevant* phrases from a document [42]. AKE is quite different from NER, as while the former is interested in finding the *small* set of *the most relevant* terms in a document, the latter is focused on finding *all the terms* of some selected types.

AKE can be performed as both an unsupervised and supervised task, and Distiller actually has its roots in an unsupervised approach [43]. However, current state-of-the-art systems use mostly a supervised approach [44], so the framework offers the possibility to use such techniques as well.

Supervised AKE is performed using a standard supervised machine-learning pipeline. The first step is generating the candidate keyphrases, using their part-of-speech tags to select certain phrasal patterns. Then, the candidate keyphrases are assigned some features, using statistical [42], linguistic [45], or semantic [46] knowledge. Finally, a machine learning algorithm is trained and then evaluated over a set of documents associated with human-assigned keyphrases.

### Ensemble system

In order to integrate Distiller with OGER together and build an effective NER system, the candidate generation phase of the former system has been replaced by OGER’s output. In fact, the original candidate generation phase of the Distiller has to be completely discarded, because it is tailored to recognizing “generic” noun phrases, which might not even be technical terms.

For this reason, in this work we follow and extend the same process we presented in [11], so the entity extraction pipeline is structured as follows:

1. Given an input document, OGER matches all the biomedical terms that appear in at least one of the selected ontologies;
2. Distiller receives the terms selected by OGER and assigns them some features, preparing them to be processed by a machine learning system;
3. A machine learning system, trained on the CRAFT corpus, selects the relevant entities in the document using the information generated in the previous steps.

The machine learning algorithms used are neural networks (NN), as they were the best performing algorithm in [11], and Conditional Random Fields (CRF), as they are currently considered the state-of-the-art algorithm, as pointed out in [24]. The architecture is slightly different for the two algorithms: In the NN case, Distiller acts as a filter on OGER’s output, i.e. it performs a binary accept/reject classification for each entity candidate. In contrast, the CRF-based version considers any token sequence in the text as an entity candidate, using OGER’s annotations only as a feature among many. Hence, the output of the NN pipeline is always a subset of OGER’s output, whereas this restriction does not hold for the CRF pipeline.

For both algorithms, training is performed using 10-fold cross validation. As in [11], in the present paper we split the corpus for training/testing purposes, so the evaluation is performed on 20 documents only. However, there are two crucial differences from [11]. Fist, in our previous work we trained a binary classifier, i.e. a single model to detect *all* entity types, while here we train a separate model for each entity type present in the CRAFT corpus. Second, here we evaluate our system not only on the named entity recognition task, i.e. considering the spans and entity types produced by our ensemble system, but we also evaluate our system on concept recognition, i.e. taking into account the concept identifiers produced by OGER.

### Features

Due to the differences of the algorithms, we used slightly different features to train NNs and CRFs. In fact, the main difference between neural networks and conditional random fields features is that the former works well with both n-ary features and continuous-valued features, while the latter works better when using n-ary features (labels) only. For this reason, some features are implemented as continuous valued in NN and as binary labels in CRF, to adapt them to the algorithm used. For example, while with NN we used a counter to determine how many uppercase characters are contained in a term, the corresponding CRF feature would be a binary label indicating the presence of uppercase characters in the token. In any case, since the library used to train CRF supports the use of numerical valued features as well, we also tried to use the exact same features used in NNs training for the CRFs training, but the resulting performance was lower than using just binary labels.

The features and the configuration used to train our algorithms are listed in Table 1. The features we selected are derived from [11] where, after a process of feature selection, the best performing feature set used information about the *shape* of the token, i.e. the number of capital letters, the number of uppercase characters, and so on, plus some *domain knowledge*, i.e. features about the affixes of the words and the presence of Greek letters inside them.

**Table 1:** Feature sets: features used by the NN and CRF (see the “Features” section for details)

	Neural network	Conditional random fields
Implementation		
Software	R [67], nnet library	CRFSuite [68]
Model parameters	1 hidden layer of size 2×(*n* *u* *m* *b* *e* *r* *o* *f* *i* *n* *p* *u* *t* *f* *e* *a* *t* *u* *r* *e* *s*), softmax output layer	Training algorithm: averaged perceptron, default epsilon, 2 words window
Input	n-grams selected by OGER	Single tokens
Features		
Candidate character count	Count	—
Candidate is all uppercase	Label yes/no	Label yes/no
Candidate is all lowercase	Label yes/no	Label yes/no
Candidate contains Greek (i.e. “alpha”, *α*)	Label yes/no	Label yes/no
Candidate contains dashes (‘-’)	Count	Label yes/no
Candidate contains numbers	Count	Label yes/no
Candidate ends with a number	Label yes/no	Label yes/no
Candidate contains capital letter not in first position	Label yes/no	Label yes/no
Candidate contains lowercase characters	Count	Label yes/no
Candidate contains uppercase characters	Count	Label yes/no
Candidate contains spaces	Count	Label yes/no
Candidate contains symbols	Count	Label yes/no
2-3 character affixes appearing in an ontology in [36]	Normalized frequency	Label yes/no
Candidate is symbol	—	Label yes/no
Candidate’s part-of-speech	—	Yes, using [69]
Candidate’s stem	—	Yes, using [70]
Candidate pre-selected by OGER	—	Yes (see the “Features” section)
Total features	36	About 2.8 million
Tagging speed *(on an Intel 4720HQ CPU)*	1286 tokens/sec	632 tokens/sec

In detail, affixes (i.e. prefixes and suffixes) are particularly useful in the biomedical domain because they are often associated with a particular meaning. For example, chemical compounds often end with “*-ide*”, like “*sodium chloride*” (the common table salt), diseases often end with “*-itis*” or “*-pathy*” (like “*arthritis*” or “*cardiopathy*”), and so on.

In order to implement this feature, we used the Bio Term Hub resource [36], and we generated four affixes lists, one each for two- and three-character prefixes and suffixes appearing in the following ontologies:

- Cellosaurus [47], developed by the Swiss Institute of Bioinformatics;
- Chemical compounds and diseases found in the Comparative Toxicogenomics Database (CTD) [48], developed by the North Carolina State University;
- Entrez Gene [19], developed by the US National Center for Biotechnology Information;
- Medical Subject Headings (MeSH) [49], developed also by the US National Center for Biotechnology Information (restricted to the subtrees “organisms”, “diseases”, and “chemicals and drugs”);
- reviewed records from the Universal Protein Resource (Swiss-Prot) [50], developed by the joint USA-EU-Switzerland consortium *UniProt*.

To weigh affixes based on their frequency, each affix *a* from a terminological resource *D* is assigned a normalized score *s*∈[ 0,1] computed in this way:

$$ s(a,D) = \frac{\text{freq}(a,D)}{\max(\{ \text{freq}(a_{1},D)\dots\text{freq}(a_{|D|},D) \})} $$

where freq(*a*,*D*) is the frequency of an affix *a* in *D*. Two weights (one prefix and one suffix) for each ontology contained in BioTermHub are used as input for the neural networks, while for CRF we use only a binary flag indicating whether the affix can be found in the selected ontology. As mentioned before, we chose to use affix information differently for CRF and NN, since after testing both binary features and weighted features on both algorithms, we determined that the former approach performed better on CRF and the latter on NN.

For CRF, we also tried to add prefixes and suffixes of each token as features, similarly to what we found in [7]. We trained several models by adding affixes of two, three, four, and five characters to each feature set, with the model increasing from ∼ 2.8 million features to ∼ 11.6 million features, but we found no significant improvement in performance with respect to using our dictionary approach only.

Unfortunately, this approach would not have been feasible when using neural networks, because it would have generated a very large set of additional features, making the network practically impossible to train.

Finally, it is also worth noting another fundamental difference between the NN and the CRF approach. While the NN receives as input only the tokens selected by OGER, the input of the CRF is composed by the whole tokenized document, and the selection of a token as a potential entity by OGER is used as a feature, as pointed out in Table 1. For this reason, CRFs are able to recognize entities that are *not* recognized by OGER, while NNs cannot do this, since they know only the portions of the document selected by the dictionary-based step, and therefore act simply as a *filter* on OGER’s output.

### Test hardware

We ran the OGER and Distiller systems on a computer equipped with an Intel i7 4720HQ quad core processor running at 2,6 GHz, 16 GB RAM and a Crucial M.2 M550 SSD. The operating system was Ubuntu 16.04 LTS.

OGER obtained a performance of 5994 tokens/second when running in single thread mode, while the Distiller system processed 1286 tokens/second when using NN and 632 tokens/second when using CRF. If necessary, the OGER system can be parallelized in a straightforward manner. Its efficiency is demonstrated also by the excellent result obtained in the recent TIPS challenge [31] (see the “OGER” section).

## Results

We examined the performance of our systems in two separate evaluations. First we evaluated the performance of NER proper, i.e. we regarded only offset spans and the (coarse) entity type of each annotation produced by each system, ignoring concept identifiers. This is a direct continuation of the work presented in [11]. Subsequently, we describe the results of a preliminary concept recognition (CR) evaluation. To this end, we augmented the ML-based output with concept identifiers taken from the dictionary-based pre-annotations, which enabled us to draw a fair comparison to previous work in CR on the CRAFT corpus.

### Named entity recognition

We present the results obtained in Table 2. They are compared with the previous version of our system, as described in [11].

**Table 2:** Comparison of the NER performance obtained in this paper with the previous version of the system [11]

System	Precision	Recall	F1
OGER 2016	0.34	0.55	0.42
OGER+Distiller 2016	0.85	0.37	0.51
OGER	0.59	**0.66**	0.62
OGER+Distiller NN	**0.86**	0.60	**0.70**
OGER+Distiller CRF	0.69	0.49	0.58
OGER+Distiller Mixed	0.87	0.63	0.73
Distiller CRF	0.71	0.47	0.58

The best values are highlighted in boldface

The best recall is obtained by the new version of the OGER system, which obtains an overall performance higher than the previous OGER/Distiller pipeline. The 66% recall score obtained by the system offers an 11% improvement over the previous version but, perhaps more importantly, the precision of the annotations is much higher, almost doubling the precision from 34 to 59%.

The higher quality of the annotations is reflected by the fact that the neural network pipeline, which received only minor improvements from the previous version, now displays a less significant drop in recall, with a score of 60%, just six point less than OGER. In the version presented in [11], the recall drop when adding the machine learning filtering stage was much higher (18%). The neural network version improves the precision score as well, with a 1% increase. This result brings the overall F1-Score to 0.70, which makes this version of the system the best performing one.

On the other hand, the combined OGER-CRF pipeline obtains a somewhat underwhelming performance, with lower precision, recall and thus F1-score when compared to the NN. Still, both models perform better than the older version, corroborating the validity of our approach.

Running the Distiller with a CRF-trained model *without* OGER’s preprocessing step, i.e. without instructing the CRF which tokens have been marked as entities according to OGER, leads to an acceptable result, with higher precision but lower recall (see Table 2, this pipeline is called “Distiller CRF”). This is unsurprising, since CRFs are known to be good systems for named entity recognition; still, using the CRF without using OGER’s dictionary would imply the important caveat that the system would not be able to associate terms with concept identifiers, but only to recognize their presence and type.

In Tables 3, 4, and 5 we analyze the performance of our system for the individual entity types. Here we consider both a *strict* evaluation, which considers correct only annotations where reference and system spans match perfectly, and a more lenient evaluation scheme, where we consider a system annotation partially correct if it overlaps just partially with a CRAFT annotation. To be precise, we used the *average* measure as defined by GATE’s Annotation Diff Tool [51], which is the mean of precision/recall/F1 in the *strict* and *lenient* measures. When a predicted annotation overlaps partially with a reference annotation, it counts both as a false positive (FP) and a false negative (FN) in the strict measure. In the lenient measure, this corresponds to a true positive (TP). Consequently, in the average measure, a partial match is counted as $\frac {1}{2}$ TP, $\frac {1}{2}$ FP, and $\frac {1}{2}$ FN, such that the denominators in precision and recall (TP+FP and TP+FN, respectively) remain constant across all three measures. If more than one predicted annotation overlaps with the same reference annotation, it is counted as a partial match only once; additional predictions are counted as false positives. The same holds for a predicted annotation overlapping with multiple reference annotations, which contribute to the false-negative count.

**Table 3:** Per-entity-type breakdown of the precision scores obtained by the different pipelines

	Evaluation method: strict			Evaluation method: average		
Entity type	OG	OG+NN	OG+CRF	OG	OG+NN	OG+CRF
All	0.59	**0.86**	0.69	0.61	**0.89**	0.80
Chemicals	0.44	**0.89**	0.48	0.45	**0.89**	0.50
Cells	0.88	0.88	**0.95**	0.93	0.94	**0.96**
Biological processes/molecular functions	0.39	**0.78**	0.68	0.45	**0.88**	0.73
Cellular components	0.51	**0.91**	0.87	0.52	**0.92**	0.90
Organisms	0.29	**0.98**	0.82	0.29	**0.98**	0.83
Proteins	0.49	**0.86**	0.74	0.50	**0.87**	0.80
Sequences	0.46	**0.89**	0.23	0.48	**0.91**	0.27

The best values are highlighted in boldface

**Table 4:** Per-entity-type breakdown of the recall scores obtained by the different pipelines

	Evaluation method: strict			Evaluation method: average		
Entity type	OG	OG+NN	OG+CRF	OG	OG+NN	OG+CRF
All	**0.66**	0.60	0.50	**0.69**	0.61	0.58
Chemicals	**0.73**	0.68	0.26	**0.75**	0.68	0.27
Cells	0.77	0.67	**0.88**	0.77	0.71	**0.89**
Biological processes/molecular functions	0.25	0.22	**0.45**	0.29	0.25	**0.49**
Cellular components	0.60	0.56	**0.67**	0.61	0.58	**0.69**
Organisms	**0.92**	0.91	0.91	**0.92**	0.91	**0.92**
Proteins	**0.84**	0.75	0.66	**0.85**	0.75	0.72
Sequences	**0.67**	0.64	0.08	**0.69**	0.65	0.09

The best values are highlighted in boldface

**Table 5:** Per-entity-type breakdown of the F1 scores obtained by the different pipelines

	Evaluation method: strict			Evaluation method: average		
Entity type	OG	OG+NN	OG+CRF	OG	OG+NN	OG+CRF
All	0.62	**0.70**	0.58	0.65	**0.72**	0.67
Chemicals	0.55	**0.77**	0.34	0.56	**0.77**	0.35
Cells	0.80	0.76	**0.91**	0.84	0.81	**0.92**
Biological processes/molecular functions	0.30	0.35	**0.54**	0.35	0.39	**0.58**
Cellular components	0.55	0.70	**0.75**	0.56	0.71	**0.78**
Organisms	0.44	**0.94**	0.87	0.45	**0.94**	0.88
Proteins	0.62	**0.80**	0.70	0.63	**0.80**	0.76
Sequences	0.54	**0.75**	0.12	0.57	**0.76**	0.13

The best values are highlighted in boldface

It is interesting to see that the NN model obtains very good F1-Scores (> 70*%*) on almost all entity types, but has some problems to identify biological processes and molecular functions. Nevertheless, the problems on this category do not hinder the performance of the general model, which is able to obtain the best precision on all categories except for cells. Unfortunately, an almost optimal precision is not always followed by a good recall, like in the previously mentioned case of biological processes and molecular functions, where we have 78% precision and only 22% recall.

The CRF model achieves good or acceptable F1-Scores for the majority of the entity types, but appears to have trouble with correctly identifying chemicals and – severely – sequences. In fact, the scores for these two entity types are so low that the overall score for the CRF pipeline is lower than OGER, even though CRF clearly beats OGER in five out of seven entity types. This partially explains the counterintuitive finding that a plain dictionary-based system achieves better results than the CRF-based system.

Using these results, we built a “mixed” system composed of the best performing models, i.e. using CRFs for cells, biological processes and molecular functions, and cellular components, and NNs for the other entity types. This model, labeled “OGER+Distiller Mixed” in Table 2, is obviously the best possible system, with a 3% increase in F1-score when compared to the NN approach; however, this is a purely academic exercise, since in practice it is very difficult to combine the NN and the CRF models due to their very different nature.

Finally, the assumption that CRFs are able to recognize entities which are *not* detected by OGER is evident by looking at the recall figures in Table 4. In fact, considering cells, biological processes and molecular functions, and cellular components, we see that the CRF pipeline improved OGER’s recall figures by 11%, 20% and 7%, respectively. This happens because for CRF we adopt a token-by-token process, where a correct item can be a token annotated by the CRAFT annotators but *not* by OGER, while in the NN pipeline the correct samples are n-grams annotated both by OGER and the CRAFT corpus annotators. Unsurprisingly, these three entity types are the ones where the CRF obtains an overall F1-score higher than NN.

### Concept recognition

The NER pipelines presented in this work are designed to perform entity annotation in terms of identifying relevant text regions *without* assigning identifiers to the recognized terms. Nonetheless, OGER performs concept recognition by default, and in the experiments reported above, the identifiers produced were simply ignored in the downstream processing and evaluation steps. For that reason, we decided to verify the potential of using the pipelines in a CR setting by carrying out an additional experiment.

We chose a simple strategy to reintroduce the concept identifiers provided by OGER into the output of the ML systems. This step was as straightforward as joining the corresponding annotations in OGER’s and Distiller’s output. For the combined OGER-CRF pipeline, this meant that CRF annotations were removed if no matching entry was found in OGER’s output.

We did not resolve ambiguous annotations; instead, multiple identifiers could be returned for the same span. While having no disambiguation at all is arguably a deficiency for a CR system, it is not imperative that each and every ambiguity is reduced to a single choice. This is particularly true when evaluating against CRAFT, which contains a number of reference annotations with multiple concept identifiers. For example, in PMID: 16504143, PMCID: 1420314, the term “fish” (occurring in the last paragraph of the “Discussion” section) is assigned six different taxonomic ranks.

Table 6 shows the performance of the described systems in a CR evaluation against CRAFT, as well as the results for a number of other systems as reported by Tseytlin et al. [24], who carried out a series of experiments using the same dataset. All figures reflect the “average” evaluation method as described in the previous section; however, at least for our systems, the difference between strict and average evaluation is so small that the numbers are the same at the given level of precision. Please note that the results reported by [24] are not perfectly comparable to the ones we obtained, since the former were tested on the *whole* CRAFT corpus, while our approach was evaluated on 20 documents only (since we used the remaining documents to train our system), as described in the “Ensemble system” section. Still, the comparison shows that even a relatively simple approach is sufficient to transform our NER pipeline into a CR system with reasonable quality. This is particularly true for the OGER-NN configuration, where both precision and recall are as good as or better than the figures for all the reported systems.

**Table 6:** Performance of the presented systems in a CR evaluation, compared to results reported in [24]

System	Precision	Recall	F1
OGER	0.32	**0.52**	0.40
OGER+Distiller NN	**0.51**	0.49	**0.50**
OGER+Distiller CRF	0.49	0.29	0.37
MMTx	0.43	0.40	0.42
MGrep	0.48	0.12	0.19
Concept Mapper	0.48	0.34	0.40
cTakes Dictionary Lookup	**0.51**	0.43	0.47
cTakes Fast Lookup	0.41	0.40	0.41
NOBLE Coder	0.44	0.43	0.43

Please note that, as stated in the “Concept recognition” section, the systems described in [24] are evaluated on the *whole* corpus, while we use 20 documents for testing and the remainder for training. The best values are highlighted in boldface

Table 7 shows the CR performance on all the considered entity types and for all the configurations of the system. Here we see that applying NN filtering after the dictionary matching results in an increment of precision in all cases except for cells, where the drop is negligible. As for the choice of ML algorithm, the NN pipeline is almost always the best performing model, winning in all entity types except for cells in terms of F1-score. However, the main difference between NNs and CRFs is that the former retains a good recall, with the worst drop of just 9% for proteins, while the latter shows a considerable drop in recall in many categories. In particular, while the CRF precision scores are generally good, if not almost optimal for some categories, the results for chemicals and sequences are very bad in terms of recall, hindering the general performance of the system.

**Table 7:** Per-entity-type breakdown of Precision, Recall, and F1 obtained by the different pipelines in the CR evaluation

	Precision			Recall			F1		
Entity type	OG	OG+	OG+	OG	OG+	OG+	OG	OG+	OG+
		NN	CRF		NN	CRF		NN	CRF
All	0.32	**0.51**	0.49	**0.52**	0.49	0.29	0.40	**0.50**	0.37
Chemicals	0.28	0.59	**0.93**	**0.61**	0.57	0.19	0.39	**0.58**	0.32
Cells	0.88	0.87	**0.98**	**0.72**	0.66	0.68	0.79	0.75	**0.81**
Biological processes/molecular functions	0.35	0.72	**0.73**	**0.19**	0.17	0.05	0.25	**0.27**	0.10
Cellular comp.	0.49	0.87	**0.89**	**0.59**	0.56	0.52	0.54	**0.68**	0.65
Organisms	0.16	**0.49**	0.47	**0.71**	0.70	0.67	0.26	**0.58**	0.55
Proteins	0.45	0.84	**0.91**	**0.83**	0.74	0.64	0.59	**0.79**	0.75
Sequences	0.27	**0.59**	0.37	**0.53**	0.51	0.06	0.36	**0.54**	0.10

The best values are highlighted in boldface

## Discussion

In this section, we analyze the results of the experiments presented in the “Results” section and we contextualize them with related work.

### Error analysis

#### NN pipeline

Since the NN output depends heavily on OGER’s input, many of its mistakes are caused by the quality of the dictionary matching. While the precision reached by this model in filtering out non-interesting terms is quite high at 85%, the majority of the errors of the NN pipelines consist in generic terms, like verbs, adverbs, and so on, that the neural network is not able to filter out. For example, in three documents, the word “error” itself is erroneously marked as an entity. Moreover, just using regular expressions to detect common suffixes and manually inspecting the results, we see that about 5% of the errors are adjectives, about 5% are adverbs, and about 9% are verbs in *-ing* form. Another typical error is constituted by common substantives marked wrongly as entities. For example, the word “region” by itself makes 4% of the errors. These errors can probably be easily eliminated by using dedicated part-of-speech features, and will eventually be tackled in future versions of the system.

The other most common category of errors are annotations that are found by OGER but not present in the CRAFT corpus. For example, in document PMID: 15917436, PMCID: 1140370, the genes “M13mp18” and “M13mp19” are detected by OGER but not in the CRAFT corpus. These errors are hard to catch with dedicated features and, on the other hand, can potentially deliver useful information, since the annotation is *conceptually* correct, so we do not consider them as a highly critical problem of our system. Nevertheless, these errors have a high impact when evaluating the model. For example, in document PMID: 16870721, PMCID: 1540739, the NN pipeline selects the protein “p53”, which is not present in the ontologies of the CRAFT corpus, but since this particular protein is central in the paper, it is repeated many times throughout the document, and its selection by the NN pipelines accounts for about 4% of the total false positives of the pipeline.

The false negatives of the NN pipeline depend largely on the false negatives of OGER, since it cannot select anything that has not been selected by OGER. Still, the NN can theoretically *remove* correct OGER selections, and analyzing the results of our system we see that this happens in practice, too. In fact, the number of the false negatives of the NN increases of about 20%, as expected by the lower recall of the NN pipeline when compared to OGER’s output. These false negatives are mostly short strings, like “BLM”, “CD21”, “p34”, with no apparent connection with the category of the word: 43% of the false negatives introduced by the NN are in fact words of 3 or 4 characters (shorter terms had been removed from the dictionary initially, as described in the “OGER” section).

#### CRF pipeline

One of the strengths of the model, i.e. detecting entities that are *not* annotated as such, is also a potential weakness while evaluating its precision. For example, in document PMID: 17696610, PMCID: 1941754, the model produces the annotation “Mei1”, which is the name of a gene/protein. This word is not annotated in the CRAFT corpus with respect to the Protein Ontology, because at the time of CRAFT’s annotation, the Protein Ontology did not include Mei1 (recent versions of the ontology do include it). CRAFT has annotations for Mei1 with respect to Entrez Gene, but this resource was ignored in our evaluation, as is described in the “CRAFT corpus” section. Differently from the false positives produced by the neural network pipeline described in the previous section, this time the concept is *not* annotated by OGER either. However, the CRF identifies that Mei1 is a protein: this is again conceptually correct, but still a mistake for the sake of evaluating the performance of the system on the CRAFT corpus.

We argue that, while these annotations hinder the *evaluation* performance of the models, they are actually desirable. This way, in fact, we are able to annotate what OGER is not able to recognize due to shortcomings in the dictionary used, thus providing information which we think is valuable for the user. Moreover, the ability of the model to select entities that are not yet present in a knowledge base is one of the desirable aspects of using a ML approach, as pointed out in the “Background” section. In our example, “Mei1” could have been a recently discovered protein which then is not yet present in the ontologies used; we argue that the ability of recognizing it as an entity and to classify its type, even *without* the ability of linking it to an actual knowledge base, would be a positive feature of our system.

On the other hand, the CRF model makes many errors where it includes punctuation in the annotation, like commas, parentheses, and so on. The relatively high frequency of this error is evident when we consider that the CRF pipeline benefits from the average evaluation (which considers partial matches as partially correct) by improving the precision score by 20%, while the NN pipeline shows a mere 3% improvement.

This model also fails to recognize certain words or their derivatives. For example, about 5% of the false negatives of the CRF pipeline consist in the failure of recognizing the word “gene” or words with the same root (like “*genes*”, “*genetic*”, “*genome*”, etc.); another 2% is due to the failure of recognizing the word “expression” or similar. Moreover, many acronyms are not recognized: in the document PMID: 15061865, PMCID: 400732, the model fails to recognize every reference to the acronym “D2R” (Dopamine D2 receptor), weighing about 2% of the *total* false negatives. The same holds for the acronyms or short names (“MLH1“ or “MLH3”, “PPAR *α*”, “PPAR *δ*”, etc.), with about 15% of the false negatives being acronyms.

A particular case are annotations where the annotation *span* is wrong. For example, in document PMID: 17425782, PMCID: 1858683, the pygopus genes “Pygo1” and “Pygo2” often appear as “Pygo1/Pygo2”; in the CRAFT corpus, the single gene names are annotated separately, while the CRF annotated the whole string. The gene is also often mentioned as “Pygo1 gene”, and here again in the CRAFT corpus it is annotated as “Pygo1”, while the CRF selects the whole phrase.

#### OGER

False negatives in the OGER output have an impact on the entire system which is much higher than the one of false positives. This is particularly true in combination with the NN postfilter, since annotations missing from the beginning remain unreachable in any subsequent step. In combination with the CRF system, where the OGER annotations are seen as one feature among many, the effect is less pronounced, since initial false positives might be corrected eventually; however, OGER’s false negatives still have a strong influence on the CRF’s decision. Therefore, this error analysis focuses on annotations that were missed by the OGER system.

A frequent cause for missing annotations are synonyms that are not covered by the dictionaries. For example, “antibody” is used in document PMID: 12925238, PMCID: 194730 for the more specific term “immunoglobulin complex”, while the Gene Ontology does not list “antibody” as a name for this (or any other) concept. Missing synonyms occurred with all entity types, and they were the predominant source of error for proteins (estimated to be more than one third of all misses). Sometimes, terms are abbreviated in text, such as “olfactory receptor” in document PMID: 14611657, PMCID: 329117, which should be “olfactory receptor activity” according to the dictionary. This was seen frequently with cellular components (more than 25%), organisms, biological processes and molecular functions (less than 10%), and proteins (around 5%). Another cause of misses are splitting and reordering of multi-word terms, as is both illustrated with “gene expression”, which is rephrased as “expression of […] gene” in the same document. Along with hyperonymy (see the “antibody” example above), splitting and reordering contributed considerably to the low recall we obtained for biological processes and molecular functions. Linguistic variation at the level of morphology was another source of mismatch between the terminologies and the texts. Derivation (changes in part-of-speech, such as “mammal”/“mammalian”, “nucleus”/“nuclear”, “gene”/“genetic”) was the most common problem for organisms (more than 25%), and also occurred among cellular components and sequences (less than 10%). Likewise, plural forms were not always mapped correctly to their singular forms listed in the dictionaries, especially in the case of acronyms (such as “cDNAs”), where stemming was disabled.

Some instances are very close misses. For example, given the dictionary spelling “PPAR-delta”, OGER was unable to capture “PPAR *δ*” in document PMID: 15328533, PMCID: 509410. Even though the matching strategy was designed to be robust against this kind of variation in terms of punctuation and transliteration, this particular case fell through the net. In order for two variants to be considered equivalent, a hyphen may only be dropped if it connects two strings with a different character class (such as alphabetic and numeric, e.g. “CRB-1” matches “CRB1”). However, a transition from one script to another (Latin →Greek) does not qualify as a token boundary on a par with a hyphen – a design decision which should be reconsidered.

### Related work

The field of named entity recognition has decades of history, with early work focusing on extracting a single entity type, such as protein names, from scientific papers [52]. Later on, some scholars started to introduce the use of terminological resources as a starting point for solving this problem [53].

The most recent state-of-the-art performance is obtained by using supervised machine-learning based systems. For extracting chemical names, [7] describes how two CRF classifiers are trained on a corpus of journal abstracts, using different features and model parameters. The output of the two classifiers is merged in different ways, attempting to combine the strengths of each method, using a-posteriori knowledge (performance on a test set) or the models’ own confidence. The approach in [8] also tackles chemical name extraction with CRF, partly using the same software basis as the previous one. The system is trained in a semi-supervised setting by adding a large collection of unlabeled abstracts and full-text documents. For tagging gene names, [54] describes another supervised sequence-labeling approach. The output of a CRF classifier is post-processed through graph propagation in order to account for unseen data occurring in the test set.

There is growing interest in hybrid machine learning and dictionary systems such as the one described in [10], which obtains interesting performance on chemical entity recognition in patent texts. The authors of [55] use different approaches for different entity types (machine learning for chemical names, dictionary-based for organism and assay entities); given the complementary application, this is not a hybrid approach in the strict sense. A contrastive overview that also covers rule-based approaches is given in [56]. While focusing on chemical entity recognition, their findings are equally applicable to other entity types.

In the field of entity linking, dictionary-based methods are predominant, since the prediction of arbitrary identifiers cannot be modeled in a generalized way. In [57], the authors explore ways to improve established information retrieval techniques for matching protein names and other biochemical entities against ontological resources. Using the CRAFT corpus, they measure the impact of case sensitivity and the information gain of individual tokens in multi-word terms. Another strategy borrowed from information retrieval for increasing the coverage of recognized entities is term expansion, i.e. indexing additional term synonyms drawn from another source of knowledge. For example, known orthologous relations can be exploited by substituting a mentioned protein with an evolutionarily and functionally equivalent protein from another species. This is applied to the detection of protein interactions in full text in [58]. The TaggerOne system [59] uses a joint model for tackling NER and linking at the same time – yet another example of a hybrid system that combines machine learning and dictionaries. Using an annotated corpus for training, the NER task is learned through a semi-Markov model, which is an adaptation of Markov models well-suited for detecting multi-word terms. For linking, the extracted terms and the dictionary entries are projected into the same vector space. Machine learning also often plays an important role when it comes to entity disambiguation. As an example, in [60] disambiguation is addressed with word embeddings, which are used for comparing the context of annotated terms with dictionary definitions of the candidate concepts.

The Colorado Richly Annotated Full Text (CRAFT) corpus [15, 61] has been built specifically for evaluating these kinds of systems. In [62] (and, with some more detail, in [63]), the authors used the corpus to evaluate several concept recognition tools, showing how they perform on the individual entity types in the corpus. Later, Tseytlin et al. [24] compared their own NOBLE coder software against other concept recognition algorithms, showing a top F1-score of 0.44. Another system that makes use of CRAFT for evaluation purposes is described in [64]. In a series of experiments including all entity types except for sequences, the authors were able to outperform existing systems in terms of F1-score, achieving approximately 93%, 75%, 78%, 60%, 54%, 44%, and 50% in an exact-match evaluation (NER, no identifiers) for species, cells, cellular components, chemicals, genes and proteins, genes, and biological processes and molecular functions, respectively.

## Conclusions

In this paper, we have presented an efficient, high-quality system for biomedical NER. We have shown that it can be easily extended to produce concept identifiers, achieving state-of-the-art results in a Concept Recognition (CR) evaluation. The presented system is a two-stage pipeline with a dictionary-based pre-annotator (OGER) and a machine-learning classifier (Distiller). In a contrastive evaluation, we examined the respective quality of the pre-annotations and two different classification approaches.

We evaluated both processing speed and annotation quality in a series of in-domain experiments using the CRAFT corpus. OGER’s scalability and efficiency was also demonstrated in the recently held TIPS task of the BioCreative V.5 challenge. For the NER performance, we compared a NN classifier, which acted as a postfilter of the dictionary annotations, to a CRF classifier that used OGER’s output as a feature among many. While the CRF pipeline showed interesting behavior by predicting terms that were missing from OGER’s dictionary, it was beaten by the NN system for the majority of the entity types and in the global evaluation, where the latter achieved a precision of 86% at a recall of 60% (F1: 70%). By augmenting the classifier output with concept identifiers from the pre-annotations, we were able to perform a CR evaluation. Again, the NN system outperformed the CRF approach with a precision of 51% at a recall of 49%, which is well in line with scores reported in related literature.

As future work, we will consider to tackle specific terminological categories where the classifiers fail to obtain good performance, like biological processes and molecular functions. Moreover, OGER performance can still be improved in terms of recall by allowing multi-word terms to be reordered or shortened, or even split apart. Also, recall can be increased by means of term expansion, e.g. by collecting additional synonyms from other sources (terminologies or corpora). In particular for biological processes and molecular functions, these two strategies can be combined to generate new term variants, as is shown in [65]. Furthermore, we consider improving the classification performance of our system on the entity types where we fail to obtain satisfactory results and, more importantly, to develop a concept disambiguation stage that is able to choose between the many concept IDs suggested by OGER. Finally, in order to better compare our work to other state-of-the-art systems, we will consider to extend the evaluation to the full CRAFT corpus by using other resources to train our system, or to other corpora, like the ShARe corpus [66] used by [24].

## Abbreviations

AKE: Automatic keyphrase extraction
API: Application programming interface
CR: Concept recognition
CRAFT: Colorado richly annotated full text corpus
CRF: Conditional random fields
ML: Machine learning
NCBI: the US National Center for Biotechnology Information
NER: Named entity recognition
NLP: Natural language processing
NN: Neural network
PMCID: PubMed Central ID
PMID: PubMed ID
TIPS: Technical interoperability and performance of annotation servers (track at the BioCreative V.5 challenge)
